# Effects of Saffron (*Crocus sativus L.*) Aqueous Extract on *In vitro* Maturation, Fertilization and Embryo Development of Mouse Oocytes

**Published:** 2011-12-22

**Authors:** Somayeh Tavana, Hussein Eimani, Mahnaz Azarnia, Abdolhossein Shahverdi, Poopak Eftekhari-Yazdi

**Affiliations:** 1. Department of Embryology, Reproductive Biomedicine Research Center, Royan Institute for Reproductive Biomedicine, ACECR, Tehran, Iran; 2. Department of Biology, Faculty of Science, Kharazmi (Tarbiat Moallem) Uuniversity, Tehran, Iran; 3.Department of Anatomy, Faculty of Medicine, Baqiyatallah (a.s.) University of Medical Sciences, Tehran, Iran

**Keywords:** Crocus sativus, Oocyte, *in vitro* maturation, Antioxidant, Mice

## Abstract

**Objective:**

Lower pregnancy rates of *in vitro* matured oocytes compared to those of in vivo stimulated cycles indicate that optimization of *in vitro* maturation (IVM) remains a challenge. Reduced developmental competence of *in vitro* matured oocytes shows that current culture systems for oocyte maturation do not adequately support nuclear and/or cytoplasmic maturation. Therefore this study evaluates the effects of different concentrations of saffron (*Crocus sativus L.*) aqueous extract (SAE), as an antioxidant agent on IVM of immature mouse oocytes.

**Materials and Methods:**

In this experimental study ,cumulus-oocyte complexes (COCs) were collected from 6-8 weeks old novel medical research institute (NMRI) female mice ovaries. COCs were cultured in IVM medium supplemented with 0 (control), 5, 10, 20 and 40 µg/ml of SAE in 5% CO_2_ at 37℃. The rates of maturation, fertilization and development were recorded. ANOVA and Duncan's protected least significant test, using the SAS program was applied for all statistical analysis.

**Results:**

The maturation rate was significantly higher in all groups treated with different concentrations of SAE compared with the control group (p<0.05). However, the lower concentrations of SAE (10 and 5 µg/ml) in maturation medium respectively increased the fertilization rate of oocytes and *in vitro* developmental competence when compared with the control group (p<0.05).

**Conclusion:**

The results of this study indicate that lower concentrations of SAE are more appropriate to be added to maturation medium when compared with other experimental and control groups. Generally, we conclude that addition of appropriate amounts of natural extracts such as SAE to maturation medium improves oocyte maturation and embryo development.

## introduction

*in vitro* growth of follicles and *in vitro* maturation (IVM) of oocytes are novel approaches to obtain mature oocytes. The first report of an IVM procedure was in 1939 (1, 2). Complete maturation of oocytes needs both nuclear and cytoplasmic maturation. Therefore, the lack of complete cytoplasmic maturation can lower the ability to form a male pronucleus and reduction in developmental competence of oocytes (3-6).

Identification of the optimal condition is the most important subject for IVM techniques used in basic agricultural and biotechnology research. Embryo physiology and viability are affected by the culture conditions; however nuclear and/or cytoplasmic maturation are not supported properly by current culture systems (7). Additionally, unfavorable media conditions in mammals leads to declines in developmental competence. Oocyte viability is reduced by the effects of heat stress, oxygen concentration and glucose content (8,9). *in vitro* culture conditions have higher concentrations of O_2_ than in vivo conditions. It has been found that higher levels of reactive oxygen species (ROS) produced through *in vitro* cultures affect and impress (10) fertilization rate, subsequent embryo development and clinical pregnancy rates (11). Both exogenous and endogenous antioxidants are utilized in the cellular defense system (12). It was reported that the addition of various antioxidants such as beta-mercaptoethanol, taurine, hypotaurine, vitamin E, and vitamin C to the culture media increased the rate of blastocyst formation (13).

It has been shown that the therapeutic properties of plants are due to their phytochemicals, which have antioxidative effects (12). *Crocus sativus L.* as a carotenoid, is a perennial stemless herb of the Iridaceae family. It is widely cultivated in Iran and other countries, including India and Greece and it is commonly known as saffron (14).

Volatile factors (safranal), bitter principles (picrocrocin) and dye materials (crocetin and its glycoside, crocin) (14) are agents of the therapeutic effects of saffron. Saffron or its active constituents have demonstrated anticonvulsant (15), antidepressant (16), anti-inflammatory, antinociceptive (17) and antitumor activities (18, 19). Radical scavenger effects as well as learning and memory-improving properties (20, 21) and promotion of the diffusion of oxygen in different tissues (14) have also been reported. In addition, the carotenoids components of saffron, known as biological antioxidants, play important roles in human health via the protection of cells and tissues from damaging effects of free radicals and singlet oxygen (22, 23) Crocin and crocetin, two main chemical components of saffron, protect cells from oxidative stress by scavenging free radicals such as superoxides (24). Since antioxidants influence oocyte maturation capacity and by considering the ability of saffron to clean free radicals, this study evaluates the effects of saffron aqueous extract (SAE) on IVM and subsequent embryo development of mouse oocytes.

## Materials and Methods

Unless indicated, all chemicals were purchased from Sigma,Germany. All procedures performed on animals received the prior approval of the Ethics Board at Royan Institute.

### Plant materials

Saffron stigmas were collected from Ghaen (Khorasan Razavi Province, Northeast Iran). The plant was authenticated and voucher specimen coded 408 was deposited at the herbarium of the Department of Pharmacognosy, Faculty of Pharmacy, Shaheed Beheshti University of Medical Sciences, Tehran, Iran.

### Preparation of the plant extract

About 10 g of stigmas were ground to a powder and dried in the shade at room temperature. Dried stigmas were decocted in water for 30 minutes. Thereafter, the extract was filtered and concentrated using a rotary evaporator apparatus (Heidolph, Germany). The final weight of the crude extract was 2 g. The SAE was maintained at 4℃ throughout the experiments. Before adding SAE to maturation medium, it was filtered by 0.22 µm filters.

### Collection of immature cumulus-oocyte complexes

Oocytes were obtained from 6-8 week old novel medical research institute (NMRI) female mice (purchased from Pasture Institute, Tehran, Iran). Animals were kept under controlled conditions (12 hours light/12 hours dark) and fed with water and pellets ad libitum. They were killed by cervical dislocation and their ovaries transferred into dissecting medium that contained minimum essential medium (MEM-α) supplemented with 5% fetal bovine serum (FBS), 100 IU penicillin and 100 IU streptomycin. Germinal vesicle-stage oocytes (GV) of the ovarian follicles were released by puncturing with a 26-gauge sterile needle under a stereomicroscope and acquired oocytes were used for the IVM procedure.

### In vitro maturation

IVM medium consisted of MEM-α supplemented with 100 IU penicillin, 100 IU streptomycin, 5% FBS, 7.5 IU/ml recombinant human follicular stimulating hormone (rhFSH; Organon, Holand) and 100 IU/ml human chorionic gonadotrophin (hCG) Organon, Holand(. Lethal dose (LD50) values of aqueous stigma extract were reported to be 6.67 g/ kg in rats (16). Therefore, the doses employed in the present study (5, 10, 20 and 40 µg/ml) were much lower than the reported LD50. Different concentrations of SAE (0, 5, 10, 20 and 40 µg/ml) were added to the maturation medium. The 10-15 COCs were transferred to 25 µl drops that were covered with mineral oil and cultured for 16-18 hours at 37℃ and 5% CO_2_. At various intervals from the onset of incubation, oocytes were observed by invert microscope (Nikon,Japan) and observation of nucleus morphological changes [GV and germinal vesicle break down (GVBD)] or the extrusion of first polar body (Metaphase II: MII) were used as the criterion for nuclear maturation of GV-stage oocytes. Oocytes with extruded first polar body (PB1) were used for *in vitro* fertilization (IVF).

### IVF and in vitro development

Epididymal sperm suspentions were prepared from 6-8 weeks old adult NMRI male mice and incubated for 1 hour in IVF medium to ensure capacitation. *in vitro* matured oocytes from each experimental group were placed in 100 µl IVF medium and 2×10^6^ sperm/ml was added to each drop. The drops were incubated for 4-6 hours at 37℃ and 5% CO_2_ in IVF medium. IVF and sperm capacitation medium consisted of T6 medium supplemented with 15 mg/ml bovine serum albumin (BSA) (equilibrated at 37℃ in 5% CO_2_). Inseminated oocytes were collected, washed and placed in 20 µl drops of IVD medium (T6 with 4 mg/ml BSA) and incubated at 37℃ and 5% CO_2_. After IVF, oocytes were monitored by invert microscope and the percentage of 2 pronucleus (PN) formation was recorded to evaluate fertilization rate. During IVD (96 hours), the numbers of 2 cell, 4 cell, morula and blastocyst embryos were recorded daily.

### Statistical analysis

ANOVA and Duncan's protected least significant test, using SAS program (Statistical Analysis System version 1.9) was used for all statistical analyses. All percentages of values were subjected to arcsine transformation prior to analysis. All data was expressed based on mean ± SEM. A probability of p<0.05 was considered statistically significant.

## Results

In this study, oocytes were cultured for 18 hours in IVM medium supplemented with various concentrations of SAE. As shown in table 1, the percentage of metaphase oocytes significantly increased in all four experimental groups compared to the control group (p<0.05). The addition of 10 µg/ml SAE extract to maturation medium significantly increased the fertilization rate compared to the group treated with 5 µg/ml and the control groups (p<0.05; Table 2).

Addition of 5 and 40 µg/ml SAE extract during IVM significantly increased the number of 2cell embryos compared to the control group. The addition of 5µg/ml SAE extract to maturation medium significantly increased the percentage of blastocysts compared to the group treated with 40 µg/ ml and the control group. Blastocyst formation decreased when 40 µg/ml SAE extract was added to the maturation medium. As shown in table 2, no significant difference was observed between groups treated with 40 µg/ml compared to the control group.

**Table 1:** Effect of SAE on IVM rates


Different concentrations (µg/ml) of SAE		Maturation stage of oocytes (%)		
	Total COCs	GV (mean ± SEM)	GVBD (mean ± SEM)	MII (mean ± SEM)
0	76	14.7 ± 2.3^a^	24.8 ± 1.6^a^	61.5 ± 2.1^b^
5	94	10.8 ± 1.4^a, b^	13.0 ± 2.2^b^	73.4 ± 3.1^a^
10	96	11.7 ± 1.4^a, b^	17.0 ± 2.6^b^	70.6 ± 2.6^a^
20	102	8.3 ± 0.4^b^	14.1 ± 1.9^b^	74.0 ± 2.4^a^
40	98	8.7 ± 0.6^b^	13.2 ± 2.0^b^	75.5 ± 2.3^a^


Percentage of metaphase **II** oocytes (**MII**), percentage of oocytes arrested at germinal vesicle (**GV**) and percentage of oocytes at germinal vesicle break down (**GVBD**) stages. All experiments were repeated eight times. Data are expressed as mean ± **SEM**. Different superscripts (a and b) show significant differences in a column and similar superscripts show no significant differences in a column (p<0.05).

**Table 2:** Effect of SAE on IVF and IVD


Different concentrations (µg/ml) of SAE	No. of matured oocytes	Fertilized ova with 2PN (%)	24 hours	96 hours
			2cell	Blastocyst
0	59	71.06 ± 2.6^b^	69.1 ± 3.6^b^	20.0 ± 2.7b^b^
5	86	71.84 ± 1.8^b^	78.5 ± 1.3^a^	29.8 ± 2.3^a^
10	87	78.85 ± 0.9^a^	75.2 ± 2.6^a, b^	28.7 ± 2.5^a, b^
20	90	74.28 ± 2.4^a, b^	74.2 ± 2.7^a, b^	27.4 ± 4.8^a, b^
40	93	75.04 ± 1.2^a, b^	77.2 ± 1.9^a^	20.5 ± 1.7^b^


Percentage of fertilized ova with 2PN (%) and percentage of embryos expressed as mean ± **SEM**. All experiments were repeated eight times. Different superscripts (a and b) indicate significant differences and similar superscripts show no significant differences in a column (p<0.05).

## Discussion

In this study the effect of saffron supplementation during IVM of mouse oocytes was assessed. The major findings of this research showed that the addition of all experimental amounts of SAE to the maturation medium improved maturation rate. While lower concentrations increased both IVF and IVD. Nair et al. observed that saffron increased the intracellular levels of reduced glutathione and suggested that saffron had antioxidant activity (25). It was demonstrated that the addition of antioxidants such as β-mercaptoethanol (26), cysteamine (27) and glutathione (28) to maturation medium positively influenced subsequent embryo development of mouse oocytes. It has been shown that saffron, a medicinal plant (29), exhibited strong radical scavenging properties. Crocin and crocetin derivatives have been reported to be the most abundant constituents of saffron (14, 18, 30). Saffron components protected cells by binding to nucleic acids, proteins and lipids (31). Crocin, a bioactive constituent of Crocus sativus, exhibited significant radical scavenging activity and thus, antioxidative activity (32). Previous studies have shown that crocin exerts *in vitro* antioxidative effects (32). Supplementation of medium by an antioxidant has been shown to remove two-cell block incidence in embryo development of mice (33, 34). In addition, the positive effects of insulin-like growth factor I, II and epidermal growth factor was reported on developmental competence of embryos exposed to exogenous oxidative stress (35). Regarding to our findings, the improved rate of *in vitro* embryo development may be due to antioxidative effects of SAE components that oocytes are exposed to them during IVM procedure. Glutathione level is considered to be an indicator of cytoplasmic maturation (36, 37). Previous reports on increased levels of glutathione through *in vitro* experiments by saffron have suggested that scavenging of free oxygen radicals is linked to glutathione, which may impact nuclear and cytoplasmic maturation of mouse oocytes (38). In several species, intracellular glutathione levels in mature oocytes help mediate sperm DNA condensation and male pronucleus formation post-fertilization (39-41). Therefore, observation of improvement in the rate of 2PN production in this research seemed to be related to the effect of saffron on glutathione levels in MII oocytes obtained after IVM. The optimum concentration of antioxidants such as amino acids and vitamins help ROS scavenging (42). Wang and co-workers have demonstrated that supplementation of appropriate concentrations of green tea polyphenols as antioxidants through maturation of bovine oocytes increased blastocyst formation. It was shown that the optimum concentration of Papaver rhoseas L. extract in maturation medium caused improvement in the rate of oocyte maturation and subsequent embryo development (43). Similarly, in this research the natural extract of saffron positively effected IVM, which was concentration dependent. All SAE concentrations increased maturation rates. However lower concentrations of SAE exhibited both higher rates of maturation and blastocyst formation. Lower IVD with the addition of a higher concentration of SAE to the maturation medium seemed to be due to the effect of a higher amount of crocetin. Previous reports have indicated that higher concentrations of crocetin exhibited lower antioxidative effects (44). Lower IVF and IVD rates when compared to the IVM rate in this research, were the same as previous research on the effects of plant extract on IVM rate (43). Therefore, lower concentrations of SAE were more appropriate to be used during IVM of mouse oocytes rather than higher doses.

To our knowledge, the present study is the first to demonstrate the beneficial effect of SAE supplementation of IVM medium on early mouse embryo development. The improving effect of natural extract on IVM has been shown in previous studies (43). The results of this research and previous research (43) indicated that the addition of those plant extracts to maturation medium as natural antioxidants was safe and possibly had lower side effects. According to findings of this experiment and previous reports (29), saffron may be regarded as a valuable plant source for use in traditional medicine.

## Conclusion

Supplementation of IVM media with optimum concentrations of antioxidants such as SAE may help increase the numbers of blastocysts obtained from the IVM-IVD procedure. The improved effects might be dependent on the SAE concentrations in maturation medium.
